# Supplementary material for: Canopy mortality has doubled in Europe’s temperate forests over the last three decades
Source: Nat Commun. 2018 Nov 26;9:4978. doi: 10.1038/s41467-018-07539-6 (PMC6255806; doi:10.1038/s41467-018-07539-6)
Supplement: Supplementary file 1 — Supplementary Information [file 41467_2018_7539_MOESM1_ESM.pdf]

Canopy mortality has doubled in Europe's temperate forests over the last three decades

Cornelius Senf, Dirk Pflugmacher, Yang Zhiqiang, Julius Sebald, Jan Knorn, Mathias Neumann, Patrick Hostert and Rupert Seidl

Supplementary Information

Supplementary Note 1

Europe's forests are currently on a recovery trajectory from past land use [1]. In addition, tree growth is widely benefiting from CO_2 fertilization, moderately warmer temperatures, and atmospheric nitrogen deposition [2]. As a result, forest age, growing stock, and individual tree dimensions are increasing in Europe's forests [3]. These changes in forest structure and demography have implications for our expectations regarding the relationship of different mortality indicators, such as canopy mortality, biomass mortality, and individual tree mortality. In order to illustrate the expected relationships between these indicators we here review the theoretical background of expected changes with increasing tree diameter (dbh, here used as joint proxy for an aging demography and an increase in tree growth).

The canopy space (*CS*) required by a tree increases as trees increase in size. The maximum crown radius of a tree (R_{max}), which can serve as a proxy of its required canopy space, is linearly related to a trees dbh [4] (Eq. 1). Consequently, the canopy space required by a tree increases proportionally with the square of its dbh (Eq. 2). Likewise, the stem biomass of a tree (*BM*) increases exponentially with dbh, with typically observed exponents varying around a value of two [5] (Eq. 3). We thus expect that canopy mortality and stem biomass mortality (which is strongly correlated to stem volume-based mortality) change at roughly the same rate as forests increase in diameter.

$$R_{max} \propto dbh \qquad \text{Eq. 1}$$

$$CS \propto dbh^2 \qquad \text{Eq. 2}$$

$$BM \propto dbh^2 \qquad \text{Eq. 3}$$

Resulting from minimum space requirements and increased resource competition, stem density (*N*) is decreasing as the mean diameter of a stand increases. The resulting mortality process is frequently referred to as self-thinning. Reineke (1933) [6] showed that the decrease in *N* over *dbh* is exponential, and that the change rate is consistent across different species at -1.605 (Eq. 4). The decrease in stem density with diameter is thus lower than the increase in biomass or required canopy space. Based on demographic and structural changes alone we would thus expect that

individual tree mortality decreases over time in Europe, and that this decrease happens at a lower rate than the related increase in canopy and biomass mortality.

$$N \propto dbh^{-1.605} \qquad \text{Eq. 4}$$

We note that these theoretical considerations assume complete homogeneity within stands, which is a poor approximation of most forest ecosystems. Also, the scaling coefficients indicated here do vary considerably across systems and species. The relationship between different mortality indicators elaborated here thus does not present a baseline for evaluating the observed mortality patterns. It rather provides a biophysically meaningful reference for analyzing and discussing differences in multi-proxy mortality changes.

Supplementary Methods 1

We evaluated the classification of mortality events into stand-replacing (SR) and non-stand-replacing (NSR) mortality events by comparing both classes against available forest cover estimates from Hansen et al. 2013 [7]. The Hansen data set is a global estimate of forest cover (ranging from 0 to 100%) for the year 2000 based on Landsat 7 data. We here compared the SR and NSR classes with respect to their differences in forest cover as estimated by Hansen et al. 2013. If our interpreter classification is meaningful we would expect the NSR class to have significantly higher forest cover after a mortality event than the SR class. To test this expectation we extracted forest cover estimates from Hansen et al. for all pixels with a mortality event in the time period 1998 to 2000 that were not longer than three years in duration (n = 280). For those pixels we compared the mean forest cover between the SR and NSR classes by means of parametric bootstrap with 1,000 replications. We further conducted the analysis at the level of individual countries to evaluate whether there were significant differences between countries.

The results show that the pixels classified as NSR by our interpreters had a significantly higher forest cover after disturbance than the SR class (Figure 1). On average, the forest cover was 39 (16 – 46) % higher after NSR mortality events, compared to SR mortality events. No significant differences between countries could be detected, indicating that our classification is consistent across countries. We thus conclude that while the exact estimation of residual forest cover fractions at the Landsat pixel level is challenging, calibration with high resolution imagery available in Google Earth allowed for a meaningful separation of SR and NSR disturbance events.

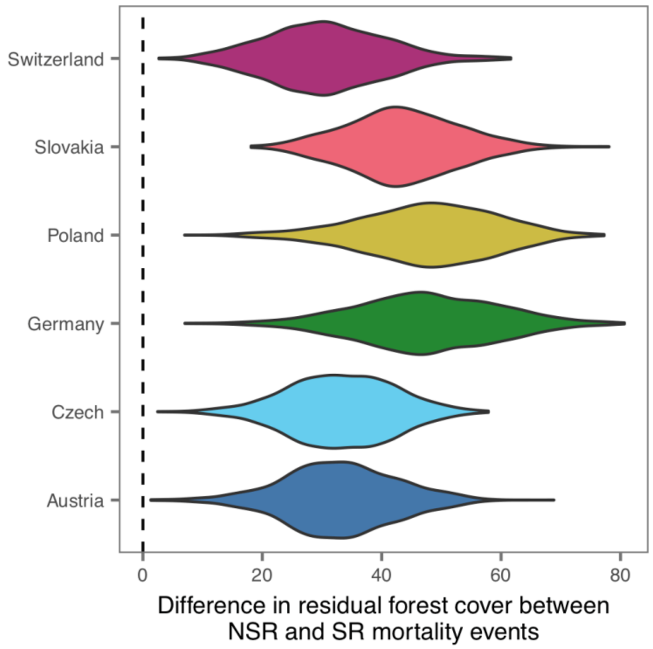

Figure 1: Differences in residual forest cover (FC) after non-stand-replacing (NSR) and stand-replacing (SR) mortality events (difference = FC_{NSR} - FC_{SR}), estimated for the year 2000 using an existing forest cover map from Hansen et al. 2013 [7]. The distributions were derived from bootstrap with 1,000 replications.

Supplementary Methods 2

We used a logit binomial model for estimating the annual log-odds of mortality, which can be back-transformed into a mortality rate ranging from zero to one using the inverse of the logit transformation. The model likelihood is specified as:

$$p(y_{[t]} | K, \theta_{[t]}) = Binomial(y_{[t]} | K, logit^{-1}(\theta_{[t]})) \quad \text{Eq. 1}$$

with $y_{[t]}$ indicating the number of mortality events in year t, K the total number of forest plots, and $\theta_{[t]}$ the log-odds of mortality for year t. A $N(\mu_{[t]}, \sigma)$ prior was put on $\theta_{[t]}$, which was re-parameterized into $\mu_{[t]} + \sigma * z_{[t]}$ with $z_{[t]} \sim N(0, 1)$ to improve sampling of the joint posterior [8]. To account for a change in mortality rate over time, we substitute $\mu_{[t]}$ by a simple linear regression over time: $\mu_{[t]} = \mu_o + \alpha * t$ with $t = \{1, \dots, N\}$. The parameter α thus estimates the change in mean log-odds of mortality over time, which can be translated into a fractional change via the exponent function. The parameter μ_o estimates the mortality rate in the initial year of the analysis (1984). We put a $N(-6, 2)$ hyperprior on μ_o, which can be seen as weakly informative as it places 95% of the prior probabilities in the interval between -7.96 and -4.04 (or a mortality rate of between 0.00 % and 1.73% yr^{-1}). A zero-truncated $N(0, 1)$ hyperprior was put on σ, and a $Student(5, 0, 0.1)$ hyperprior was put on α following recommendations given in [9]. The model is available as R package under: https://zenodo.org/record/1221340

Supplementary Methods 3

We used Bayesian log-linear mixed models to regress within country trends in mortality rates over four covariates. The model thus estimates the strength and direction of relationship between trends in mortality rates and trends in each covariate within each country, while accounting for random variation among countries. For doing so, we use a hierarchical model formulation formalized as:

$$\log(y_{ij}) = (\beta_0 + u_{oj}) + (\beta_1 + u_{1j}) * x_{ij} + \varepsilon_{ij} \qquad \text{Eq. 1}$$

with

$$\epsilon_{ij} \sim N(0, \sigma_y^2)$$

$$\begin{pmatrix} u_{oj} \\ u_{1j} \end{pmatrix} \sim MVN(0, \Sigma_u)$$

The response y_{ij} represents the mortality rate for country j and time period i, the parameter β_0 is the mean intercept, the parameter β_1 is the mean slope, x_{ij} is the covariate for each country and time period, and ${\sigma_y}^2$ is the residual variance. The higher-order parameters u_{0j} and u_{1j} define the country-specific deviation in the intercept and slope from the mean estimate, with variation expressed as a multi-variate normal distribution with co-variance matrix Σ_u, which is expressed as: $\Sigma_u = \begin{pmatrix} {\sigma_{u_0}}^2 & \rho_u {\sigma_{u_0}}^2 {\sigma_{u_1}}^2 \\ \rho_u {\sigma_{u_0}}^2 {\sigma_{u_1}}^2 & {\sigma_{u_1}}^2 \end{pmatrix}$, with ρ_u expressing correlation between ${\sigma_{u_0}}^2$ and ${\sigma_{u_1}}^2$. Hence, the formulation allows for estimating the average relationship between mortality trends and trends in covariates (e.g., temperate, growing stock) over time, while accounting for random variations in intercepts (mean mortality rate given mean covariate) and slopes among countries.

Supplementary Figures

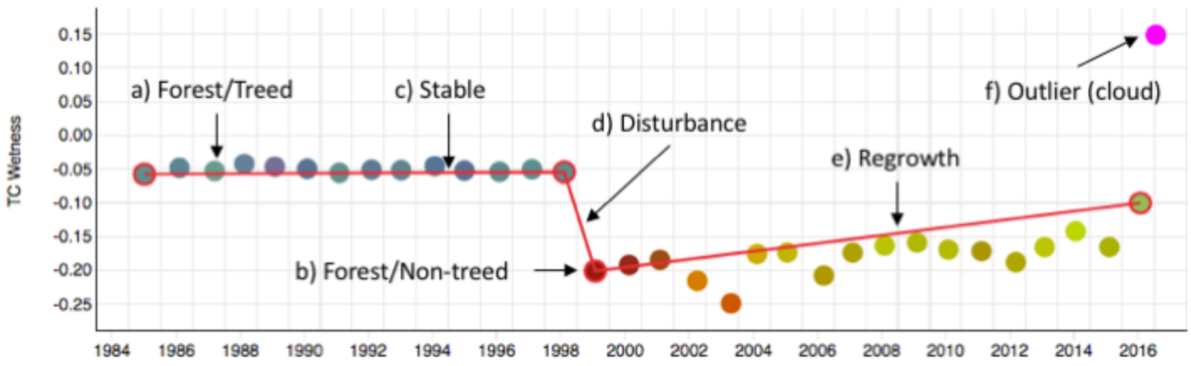

Supplementary Figure 1: Example of a Landsat time series in TimeSync. The dots indicate annual Landsat observations in Tasseled Cap space. The y-axis value is the Tasseled Cap wetness value, which serves as a proxy of canopy cover. The time series is segmented into three linear segments, presenting stable conditions, declining conditions (mortality) caused by a disturbance event, and increasing conditions (regrowth). Each segment is defined by two vertices, at which the land use and land cover are recorded. Outliers (clouds) are easily spotted by their very bright color (see year 2017).

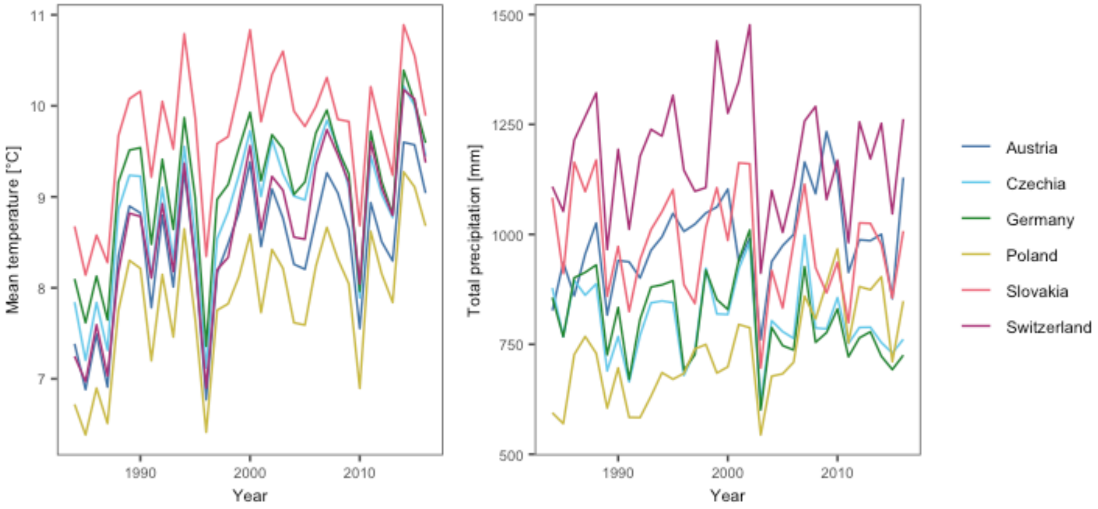

Supplementary Figure 2: Climate variables. Temperature is given as the mean annual temperature. Precipitation is given as the total annual precipitation.

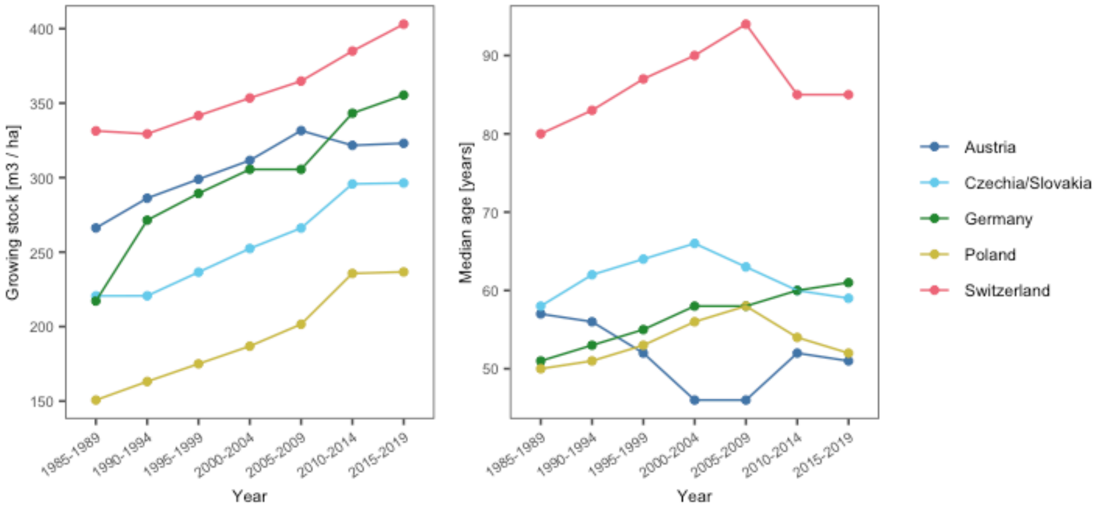

Supplementary Figure 3: Forest structural attributes. Growing stock is the live timber volume per unit area. Median age is the median of a countries age-class distribution.

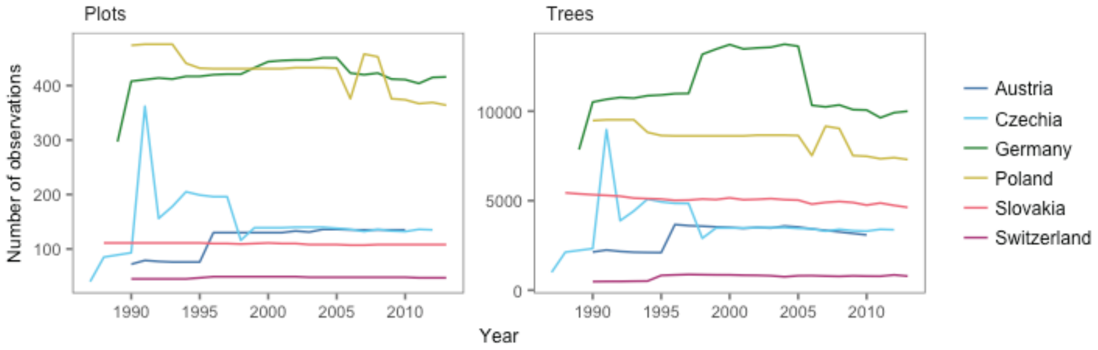

Supplementary Figure 4: ICP sample sizes given in number of plots (left) and number of trees (right). The beginning of the ICP survey varied among countries and only years with all countries having data were included in our analysis.

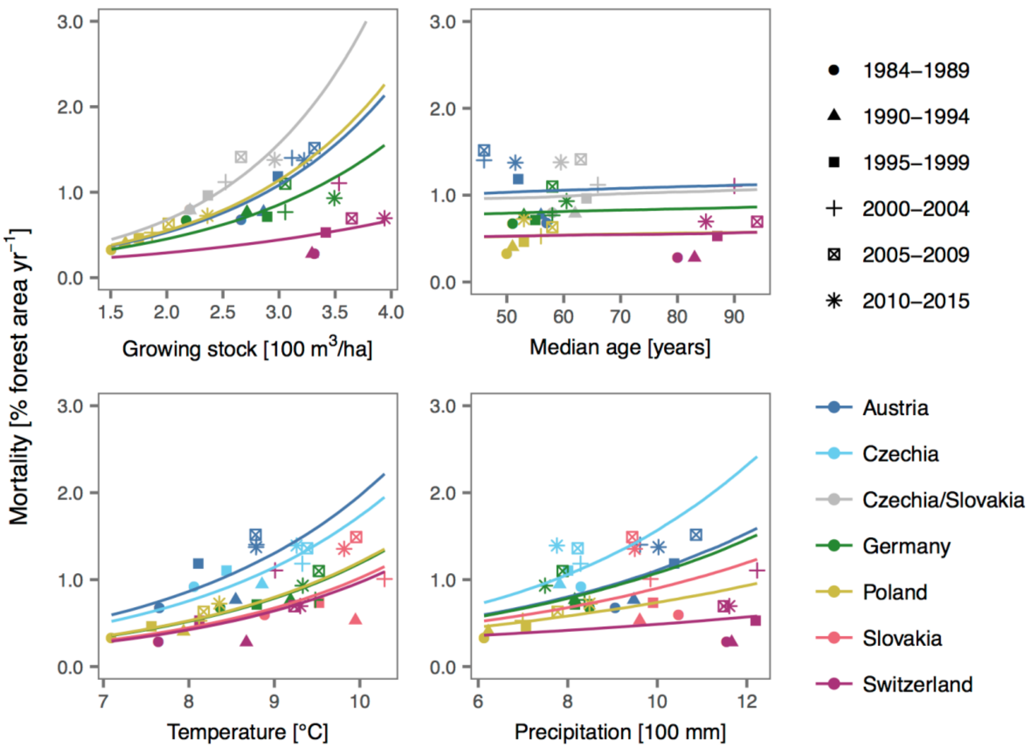

Supplementary Figure 5: Country-specific regression lines derived from the log-linear mixed effect model. For growing stock, temperature and precipitation both the intercept and slope vary by country. For median age only the intercept varies.

Supplementary Tables

Supplementary Table 1: Summary of the countries included in the study.

Country	Land area in km^2	Forest area in km^2 (% of land area)	Major forest types according to the EEA forest type classification (see legend on next page[1])
Austria	83,858	39,580 (47.2)	2, 3, 5, 6, 7, 12
Czechia	78,866	26,814 (34.0)	2, 3, 4, 5, 6, 7, 12, 13, 14
Germany	357,168	114,294 (32.0)	2, 3, 4, 5, 6, 7, 12, 13, 14
Poland	312,685	90,053 (28.8)	2, 3, 4, 5, 6, 7, 12, 13, 14
Slovakia	49,036	20,007 (40.8)	2, 3, 4, 5, 6, 7, 8, 14
Switzerland	41,290	13,130 (31.8)	2, 3, 5, 6, 7, 8, 14

[1]European Environmental Agency (2006). European forest types -Categories and types for sustainable forest management reporting and policy. EEA Technical Reports. Copenhagen, European Environmental Agency.

1. Boreal forest
2. Hemiboreal forest and nemoral coniferous and mixed broadleaved-coniferous forest
3. Alpine coniferous forest
4. Acidophylous oakwood and oak-birch forest
5. Mesophytic deciduous forest
6. Lowland to submountainous beech forest
7. Mountainous beech forest
8. Thermophilous deciduous forest
9. Broadleaved evergreen forest
10. Coniferous forests of the Mediterranean, Anatolian and Macaronesian regions
11. Mire and swamp forest
12. Floodplain forest
13. Non-riverine alder, birch or aspen forest
14. Plantations and self-sown exotic forest

Supplementary Table 2: Response design.

Vertex	Land use	Description
	Forest	The pixel is planted or naturally vegetated and has the potential to contain 10% or greater tree cover at some time during a near-term successional sequence. In accordance with FAO's definition of forest, trees (or the ground area with potential for trees) must be a minimum of 0.5 ha in size (i.e. approximately >= 6 contiguous Landsat pixels) and at least 20 m wide (i.e. less than 1 Landsat pixel). Patches of 6 or more adjoining pixels can take any shape including linear.
	Other	All pixels not meeting the forest land use definition. The land use 'Other' is also used if trees occupy a majority of the pixel area, but the criteria of the forest definition are not met (e.g., minimum size of 0.5 ha).
	Land cover	
	Treed	The pixel is comprised of at least 10% live trees.
	Non-treed	The pixel is comprised of less than 10% live trees. Might be covered by impervious, barren, grass/forb/herb, crop, snow/ice, or water.
Segment	Change process	
	Stable	No forest canopy cover change and thus no substantial change in TC wetness (and similar spectral indices).
	Mortality	Change in canopy cover due to harvest, silvicultural treatments, insects/pathogens, wind, fire, salvage of disturbed trees, avalanches, or hydrological processes. Changes in canopy cover must result in a visible decrease in TC wetness (or similar spectral indices) to be included in the analysis.
	Regrowth	Increasing canopy cover after mortality, identifiable through an increasing spectral trajectory in TC wetness.

Supplementary Table 3: Estimates of canopy mortality, wood removal, individual tree mortality, and natural disturbances. Estimates and trends are given in terms of the median and 95% credible interval. Please note that due to changes in data acquisition in 1993 (dissolution of Czechoslovakia) harvest rates for Czechia and Slovakia were combined. For comparability, we also reported combined estimates for forest mortality and individual tree mortality for these countries.

	Average mortality rate [% yr^{-1}]	Fractional change [% yr^{-1}]
Canopy mortality rates		
All	0.79 (0.75 - 0.83)	2.37 (1.38 - 3.34)
Austria	1.17 (1.09 - 1.26)	2.81 (1.11 - 4.53)
Czechia	1.17 (1.07 - 1.27)	1.76 (0.54 - 3.10)
Czechia/Slovakia	1.10 (1.03 - 1.17)	2.65 (1.66 - 3.70)
Germany	0.84 (0.75 - 0.93)	1.46 (-0.58 - 3.47)
Poland	0.53 (0.46 - 0.60)	2.99 (1.48 - 4.67)
Slovakia	0.98 (0.90 - 1.06)	4.06 (2.39 - 5.90)
Switzerland	0.61 (0.53 - 0.69)	3.60 (0.34 - 6.98)
Wood removal rates		
All	1.63	1.40 (0.57 - 2.29)
Austria	1.57	0.93 (0.10 - 1.76)
Czechia/Slovakia	1.89	1.50 (0.74 - 2.25)
Germany	1.54	1.17 (0.06 - 2.25)
Poland	1.77	2.31 (1.49 - 3.18)
Switzerland	1.22	0.16 (-0.82 - 1.10)
Individual tree mortality rates		
All	0.27 (0.25 - 0.28)	-1.45 (-4.31 - 1.36)
Austria	0.16 (0.13 - 0.20)	6.3 (-0.91 - 12.83)
Czechia	0.35 (0.31 - 0.40)	-8.5 (-13.61 - -3.07)
Czechia/Slovakia	0.37 (0.35 - 0.41)	-6.04 (-9.13 - -2.62)
Germany	0.16 (0.14 - 0.18)	0.91 (-5.00 - 6.81)
Poland	0.30 (0.27 - 0.32)	3.84 (-3.71 - 10.36)
Slovakia	0.39 (0.35 - 0.44)	-4.12 (-8.15 - 0.42)
Switzerland	0.79 (0.63 - 0.97)	-7.66 (-15.37 - 0.6)
Natural disturbances		
Bark beetle	0.09	0.02 (-0.02 – 0.05)
Wind	0.18	-0.01 (-0.05 – 0.04)

Supplementary References

1 Bebi, P. *et al.* Changes of forest cover and disturbance regimes in the mountain forests of the Alps. *Forest Ecology and Management* **388**, 43-56, doi:10.1016/j.foreco.2016.10.028 (2017).

2 Pretzsch, H., Biber, P., Schutze, G., Uhl, E. & Rotzer, T. Forest stand growth dynamics in Central Europe have accelerated since 1870. *Nature Communications* **5**, 4967, doi:10.1038/ncomms5967 (2014).

3 Forest Europe. State of Europe's forests 2015. (Ministerial Conference on the Protection of Forests in Europe, Madrid, 2015).

4 Purves, D. W., Lichstein, J. W. & Pacala, S. W. Crown plasticity and competition for canopy space: a new spatially implicit model parameterized for 250 North American tree species. *PLoS One* **2**, e870, doi:10.1371/journal.pone.0000870 (2007).

5 Zianis, D., Muukkonen, P., Mäkipää, R. & Mencuccini, M. Biomass and stem volume equations for tree species in Europe. *Silva Fennica Monographs* **4** (2005).

6 Reineke, L. H. Perfecting a stand-density index for even-aged forests. *Journal of Agricultural Research* **46**, 627-638 (1933).

7 Hansen, M. C. *et al.* High-resolution global maps of 21st-century forest cover change. *Science* **342**, 850-853, doi:10.1126/science.1244693 (2013).

8 Monnahan, C. C., Thorson, J. T. & Branch, T. A. Faster estimation of Bayesian models in ecology using Hamiltonian Monte Carlo. *Methods in Ecology and Evolution* **8**, 339-348, doi:10.1111/2041-210x.12681 (2017).

9 Gelman, A., Carlin, J. B., Stern, H. S. & Rubin, D. B. *Bayesian data analysis*. Vol. 2 (Chapman & Hall/CRC Boca Raton, FL, USA, 2014).
