# Supplementary material for: Canopy mortality has doubled in Europe’s temperate forests over the last three decades
Source: Nat Commun. 2018 Nov 26;9:4978. doi: 10.1038/s41467-018-07539-6 (PMC6255806; doi:10.1038/s41467-018-07539-6)
Supplement: Supplementary file 3 — Reporting Summary [file 41467_2018_7539_MOESM3_ESM.pdf]

nature research

Corresponding author(s): Cornelius Senf

Reporting Summary

Nature Research wishes to improve the reproducibility of the work that we publish. This form provides structure for consistency and transparency in reporting. For further information on Nature Research policies, see Authors & Referees and the Editorial Policy Checklist.

Statistical parameters

When statistical analyses are reported, confirm that the following items are present in the relevant location (e.g. figure legend, table legend, main text, or Methods section).

n/a	Confirmed	
☒	☐	The exact sample size (n) for each experimental group/condition, given as a discrete number and unit of measurement
☒	☐	An indication of whether measurements were taken from distinct samples or whether the same sample was measured repeatedly
☒	☐	The statistical test(s) used AND whether they are one- or two-sided<br>*Only common tests should be described solely by name; describe more complex techniques in the Methods section.*
☒	☐	A description of all covariates tested
☒	☐	A description of any assumptions or corrections, such as tests of normality and adjustment for multiple comparisons
☐	☒	A full description of the statistics including central tendency (e.g. means) or other basic estimates (e.g. regression coefficient) AND variation (e.g. standard deviation) or associated estimates of uncertainty (e.g. confidence intervals)
☒	☐	For null hypothesis testing, the test statistic (e.g. F, t, r) with confidence intervals, effect sizes, degrees of freedom and P value noted<br>*Give P values as exact values whenever suitable.*
☐	☒	For Bayesian analysis, information on the choice of priors and Markov chain Monte Carlo settings
☐	☒	For hierarchical and complex designs, identification of the appropriate level for tests and full reporting of outcomes
☒	☐	Estimates of effect sizes (e.g. Cohen's d, Pearson's r), indicating how they were calculated
☐	☒	Clearly defined error bars<br>*State explicitly what error bars represent (e.g. SD, SE, CI)*

Our web collection on statistics for biologists may be useful.

Software and code

Policy information about availability of computer code

Data collection	We used TimeSync (http://timesync.forestry.oregonstate.edu/) to collect data. TimeSync is described in detail in Cohen, W. B., et al. (2010). Detecting trends in forest disturbance and recovery using yearly Landsat time series: 2. TimeSync — Tools for calibration and validation. Remote Sensing of Environment 114(12): 2911-2924.
Data analysis	The data was analyzed in R (version 3.5.1). All code is available under https://zenodo.org/record/1453351 [DOI: 10.5281/zenodo.1453350]. The model used for estimating annual mortality rates and trends in mortality rates is available as R package under https://zenodo.org/record/1221340 [DOI: 10.5281/zenodo.1221339].

For manuscripts utilizing custom algorithms or software that are central to the research but not yet described in published literature, software must be made available to editors/reviewers upon request. We strongly encourage code deposition in a community repository (e.g. GitHub). See the Nature Research guidelines for submitting code & software for further information.

Data

Policy information about availability of data

All manuscripts must include a data availability statement. This statement should provide the following information, where applicable:

- Accession codes, unique identifiers, or web links for publicly available datasets
- A list of figures that have associated raw data
- A description of any restrictions on data availability

All data used in this study are deposited under https://zenodo.org/record/1453351 [DOI: 10.5281/zenodo.1453350].

Field-specific reporting

Please select the best fit for your research. If you are not sure, read the appropriate sections before making your selection.

☐ Life sciences ☐ Behavioural & social sciences ☒ Ecological, evolutionary & environmental sciences

For a reference copy of the document with all sections, see nature.com/authors/policies/ReportingSummary-flat.pdf

Ecological, evolutionary & environmental sciences study design

All studies must disclose on these points even when the disclosure is negative.

Study description	We collected land use/land cover and forest mortality information by human interpretation of satellite image trajectories.
Research sample	We applied a random sampling design, sampling 6,000 satellite pixels per country. The total sample size was 24,000.
Sampling strategy	The sample size was estimated using an existing forest cover change product with the goal of balancing estimation precision and work load.
Data collection	The data was recorded using TimeSync and followed a standardized response design in the methods section. A total of four interpreters collected the data.
Timing and spatial scale	The data was collected across six countries in Central Europe (Austria, Czechia, Germany, Poland, Slowakia, Switzerland) over the time period January 2017 to March 2018.
Data exclusions	No data was excluded.
Reproducibility	Not applicable.
Randomization	Not applicable.
Blinding	Not applicable.

Did the study involve field work? ☐ Yes ☒ No

Reporting for specific materials, systems and methods

Materials & experimental systems

n/a	Involved in the study
☒	☐ Unique biological materials
☒	☐ Antibodies
☒	☐ Eukaryotic cell lines
☒	☐ Palaeontology
☒	☐ Animals and other organisms
☒	☐ Human research participants

Methods

n/a	Involved in the study
☒	☐ ChIP-seq
☒	☐ Flow cytometry
☒	☐ MRI-based neuroimaging
